# Modelling and Optimization for Mortar Compressive Strength Incorporating Heat-Treated Fly Oil Shale Ash as an Effective Supplementary Cementitious Material Using Response Surface Methodology

**DOI:** 10.3390/ma15196538

**Published:** 2022-09-21

**Authors:** Marsail Al Salaheen, Wesam Salah Alaloul, Ahmad B. Malkawi, Jorge de Brito, Khalid Mhmoud Alzubi, Abdulnaser M. Al-Sabaeei, Mohamad Sahban Alnarabiji

**Affiliations:** 1 Department of Civil and Environmental Engineering, Universiti Teknologi PETRONAS, Bandar Seri Iskandar, Tronoh 32610, Perak, Malaysia; 2 Department of Civil Engineering, Faculty of Engineering Technology, Al-Balqa Applied University, Amman 11134, Jordan; 3 Department of Civil Engineering, Architecture and Georesources, Universidade de Lisboa, Av. Rovisco Pais, 1049-001 Lisbon, Portugal; 4 Department of Civil Engineering, Faculty of Engineering, Thamar University, Thamar 87246, Yemen; 5 Centre for Advanced Material and Energy Sciences, Universiti Brunei Darussalam, Gadong BE1410, Brunei

**Keywords:** fly oil shale ash, cement replacement, XRD, XRF, RSM, compressive strength, strength activity index, heat treatment

## Abstract

Fly oil shale ash (FOSA) is a waste material known for its pozzolanic activity. This study intends to investigate the optimum thermal treatment conditions to use FOSA efficiently as a cement replacement material. FOSA samples were burned in an electric oven for 2, 4, and 6 h at temperatures ranging from 550 °C to 1000 °C with 150 °C intervals. A total of 333 specimens out of 37 different mixes were prepared and tested with cement replacement ratios between 10% and 30%. The investigated properties included the mineralogical characteristics, chemical elemental analysis, compressive strength, and strength activity index for mortar samples. The findings show that the content of SiO_2_ + Al_2_O_3_ + Fe_2_O_3_ was less than 70% in all samples. The strength activity index of the raw FOSA at 56 days exceeded 75%. Among all specimens, the calcined samples for 2 h demonstrated the highest pozzolanic activity and compressive strength with a 75% strength activity index. The model developed by RSM is suitable for the interpretation of FOSA in the cementitious matrix with high degrees of correlation above 85%. The optimal compressive strength was achieved at a 30% replacement level, a temperature of 700 °C for 2 h, and after 56 days of curing.

## 1. Introduction

Oil is a limited and quickly depleting fossil resource whose sustainability is one of the main global concerns [1,2]. It is agreed worldwide that the era of low-cost oil has passed, and a new and unusual phase is to be faced [3]. The potential for the recovery and diffusion of unconventional resources (such as shale oil and biofuels) becomes of increasing interest and a director for recent enhancement and development. Shale oil is unconventional fuel produced from fragmented oil shale (OS) rocks with organic matter accounting for 30–35% of the total and inorganic matter (such as sandy-clay minerals) accounting for 65–70% [4,5].

There are over 600 known deposits of Shale Oil in 33 countries worldwide. The world’s shale oil deposits in all continents of the world are estimated to produce over six trillion barrels of shale oil, which is near four times the recent world resources of conventional crude oil [6,7]. Globally, the total daily production of shale oil is about 30,000 barrels, produced by Australia, Brazil, China, and Estonia [8]. The strategic value of shale oil deposits varies from country to country. For small countries with no other liquid hydrocarbon resources and high oil demand (such as Estonia, Jordan, and Morocco), the development of such resources is of vital importance strategically [8]. For some of these countries, electricity production is heavily dependent on the combustion of shale oil.

Two OS combustion technologies are frequently used for oil extraction: pulverized firing combustion method (PF) and the circulating fluidized-bed combustion method (CFB) [9]. CFB combustion technique is based on low-temperature combustion, with combustion temperatures ranging from 700 to 850 °C. PF combustion technique, on the other hand, is high-temperature combustion in which the temperature of the burning flame of pulverized oil shale exceeds 1500 °C [10]. These techniques result in three types of pollutants; gaseous, liquid, and solid substances, which need preventive requirements for disposal [11].

Due to shale oil combustion, a pronounced amount of ash is produced. This ash is one of the main obstacles that cannot be neglected when analyzing the environmental impact, damping preparation, and cost estimation [12,13,14]. The solid by-product can be divided into fly ash and bottom ash. Bottom oil shale ash (BOSA) comprises angular particles that vary in size from sand to gravel. These particles can be used as a source of aggregate in construction [15,16,17,18]. FOSA comprises finer particles with silt size. These particles are usually collected by electrostatic precipitation of combustion emissions and can be used as a filler for partial or full replacement of cement [19,20]. The amount of solid waste, i.e., oil shale ash (OSA), ranges from 40 to 80% by weight of the raw materials [21,22,23,24]. In Estonia, 10 million tons of OS are employed in the production of electricity generating about six to eight million tons of OSA annually [25,26,27,28]. In Jordan, the working deposits are estimated to produce overall more than 7 billion tons of oils resulting in a large amount of OSA that may research up to 50 billion tons in total [29,30,31,32]. Jordanian OS is high quality and shallow so mining is more efficient [33]. Therefore, rational use of the produced OSA is highly required.

OSA can be considered a plentiful and cheap material from an economic point of view. On the other side, dumping this by-product material in landfills will lead to several environmental issues and land wasting [23,34]. Therefore, action plans must be prepared to manage the growth of the shale oil production industry and these plans should consider the sustainability prospect. One of the major fields that such plans should consider is the construction sector [35,36]. This sector is a major consumer of natural resources, where the quarrying processes of aggregate and cement are considered to be responsible for several environmental issues [36,37,38]. Therefore, priority should be given to finding alternatives and replacements for these materials. Considering the utilization of OS wastes in the construction sector can provide an efficient solution serving the aforementioned purposes.

The primary chemical components of oil shale wastes are SiO_2_, Al_2_O_3_, and Fe_2_O_3_, which form about 70% of their chemical composition, which complies with ASTM requirements for natural pozzolan and fly ash to be used in concrete [39,40]. This urged the researchers to investigate the cementitious properties of OSAs and their utilization as cement replacement materials. Early research on using FOSA in concrete concluded that the original form of FOSA has low pozzolanic features and recommended that not higher than 20% by volume of cement can be replaced in mortar or concrete production [41]. Likewise, similar values can be found in several studies that explored the utilization of FOSA in self-compacting concrete, whereas 10% cement replacement provided the maximum compressive strength, high flowability, and resistance against segregation [42]. Nevertheless, a cement replacement of up to 50% could still be feasible for other structural applications [43,44,45]. In addition, some of the other researchers employed a large quantity of FOSA in mortar and concrete preparation. Using FOSA up to 50% in partial substitution of cement to produce non-autoclaved aerated concrete is worth mentioning [46,47]. The drop in strength of the produced concrete was offset by increasing the curing temperature, but water demand increased because of the binder amount [46]. Another limitation that should be investigated is the high lime (CaO) content in OSAs, CaO and mixing water react and produce calcium hydroxide (Ca(OH)_2_) within the matrix of hardened concrete. This reaction may result in the expansion and cracking of hardened concrete [20], although the micro-quartz content in FOSA suppresses alkalis by the formation of non-expanded products from the pozzolanic reactions [45]. Several studies have investigated the use of FOSA-based mortar at natural sand to binder ratio of 3:1, water to binder ratio of 0.3–0.5, and FOSA replacement levels of 7–30%. The produced mortars provided a 28 day compressive strength in the range of 34 to 45 MPa [48,49]. The obtained high strength value was attributed to the development of hydration products, especially C-S-H.

Response surface methodology (RSM) is one of the greatest efficient methods in the design of experiments (DOE) and improves their predictability and reliability while minimizing the required efforts [50]. Applications of RSM in concrete manufacturing and development are established and well known [51]. RSM was used to achieve multi-objective optimization of several concrete materials such as (i) production of masonry bricks using lightweight concrete based on micro-fines stone sludge [52], (ii) optimization of the content of binder, cooling temperature, and curing age to produce fly ash-based geopolymer mortar [53], (iii) comparing the corrosion resistance of concrete mortars using dioctyl terephthalate or polyethylene terephthalate [54] and (iv) examining the joining effect of nano-silica and crumb rubber [55,56].

The existing body of knowledge reveals that FOSA can be used as cement replacement material at small replacement ratios. On the other hand, most of the conducted research investigated the use of FOSA in its raw state without applying any considerable treatment. The FOSA raw material is usually burned at a temperature of 500–1000 °C, but no information on the burning time and exact temperature is provided [48,49,57,58,59]. Yet, the burning temperature profile should be controlled to ensure the best pozzolanic behavior [40]. There is hardly any literature on the exclusive use of OS to produce ash under controlled conditions. Therefore, this study is intended to increase the cement replacement ratios in FOSA-based mortar. To achieve this target, a systematic treatment was conducted to improve the FOSA cementitious properties. Grinding and heat treatment were applied at different levels to determine the optimum temperature and time required to produce the treated version of FOSA, considering the chemical, physical, and pozzolanic characteristics. Furthermore, studies are carried out to determine the effect of the treated FOSA on the strength and properties of cement mortar comprising a high volume of FOSA. This study not solely contributes to environmental waste minimization but also highlights the mineralogical features, chemical composition, and mechanical properties at which FOSA could be processed in a sufficient way to facilitate its use in larger quantities for mortar production.

## 2. Materials and Methods

### 2.1. Materials

FOSA was acquired from the central part of Jordan and its specific gravity was measured to be 3.2. Ordinary Portland cement (OPC) that meets the ASTM C150 [60] standard and had a 3.15 specific gravity. The natural sand used had a specific gravity of 2.65, fineness modulus of 3.1, and its particle size ranges from 0.3 to 0.85 mm.

### 2.2. FOSA Treatment

FOSA was dried for 24 h in an oven at 105 ± 5 °C to remove moisture. To remove coarser particles, the dried FOSA was sieved using a 150 m sieve. Next, the FOSA was ground to an appropriate fineness by a laboratory ball mill for 1 h at 45 rpm speed, to raise the efficiency of the succeeding thermal treatment by reducing the particles’ size and expanding their reactivity. After the milling processes, the specific surface area became around 2700 cm^2^/g with a 3.2 μm average particle size. Table 1 lists the chemical compositions of FOSA and cement.

**Figure 1:**
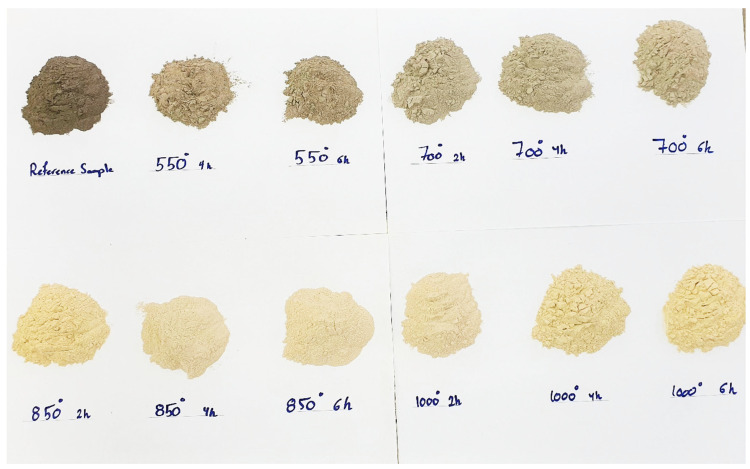
Difference in color of raw and treated FOSA at selected calcination temperatures and durations.

The temperature and duration limits simulate and summarize the parameters that were repeated in previous studies to determine their effect on this FOSA [46,57,58,59]. Lastly, the thermal-treated FOSA was sieved again to ensure the size of particles and remove the coarser and agglomerated materials using a 200 µm sieve. The change in color of the treated FOSA is displayed in Figure 1. For materials characterization, X-ray diffraction (XRD) was used to analyze the mineralogical phases. A X-ray fluorescence spectrometer (XRF) was used to measure the chemical composition of the materials.

### 2.3. Material Characterization Methods

The chemical composition (% by weight) of OPC, raw and treated FOSA was obtained by XRF, conducted using a Bruker AXS: S4 PIONEER X-ray spectrometer. The mineralogical phases of the materials were determined using a Bruker AXS: D4 ENDEAVOR diffractometer and the data were collected from 10° to 70°, at a 1° angular step using a tube with Cu anode, and a divergence slit of 1 mm. The identification of phases was achieved by comparing the obtained pattern with the reference database patterns of the international center of diffraction data (ICDD) from the DIFFRAC plus software. The crystallinity index of the studied samples was calculated with an equation based on the XRD patterns using the ORIGIN PRO software version 2021.

### 2.4. Mortar Preparation and Mechanical Properties Test

A total of 333 cubes were cast from 37 mortar mixes including the reference mix, which was designated with (0%) FOSA and prepared to have a normal consistency according to ASTM C187 [61]. The remaining 36 mixes were prepared by including the untreated (raw) and treated FOSA at three replacement levels, i.e., 10%, 20%, and 30%. These amounts and ratios developed the greatest results in the previous researches [42,48,49,62]. All ingredients were mixed as required according to the ASTM C305 procedures [63]. The produced mix was cast in 50 mm test cubes and tamped in two layers as compaction.

The cubes were cured in the molds for one day before being removed and fully immersed in water until evaluated for compressive strength at various curing ages. The compressive strength test was performed in three 50 mm cubic molds at 7, 28, and 56 days for each treatment level. The molds were tested using an automatic concrete compression device with 3000 kN capacity at a loading rate of 1 kN/s, based on the ASTM C109 specification [64]. For each mix, nine specimens were cast for the compressive strength test at 7, 28, and 56 days.

The mixes were designated by the replacement percentage followed by the abbreviation of the treatment conditions where the temperature assigned (1, 2, 3 and 4) that represent (550 °C, 700 °C, 850 °C, 1000 °C), respectively, and A, B and C represent the calcination duration (for 2, 4 and 6 h, respectively). For example, (10%, 2-B) indicates the mix in which the cement was replaced at 10% of FOSA calcined at 700 °C for 4 h. The mix proportions were kept constants for all mixes and the only variable was the replacement level for comparison purposes. The pozzolanic activity of the FOSA has been determined based on compressive strength as stated by ASTM C311 [65].

### 2.5. Data Analysis, Modelling, and Optimization Using RSM

RSM is an effective optimization tool that could be run using wide types of models, such asuser-defined, factorial design, fractional factorials, optimal mix, central composite, and historic data. The selection of the best model is affected by the existing data and the levels of the variables [50]. Linear or higher-degree polynomials functions are the formulations for the numerical models, linking the dependent and independent variables. The first-order function is expressed in the linear model shown in Equation (1), where y is the modelled response, β_o_ is the y-intercept at X_1_ = X_2_ = 0, while β_1_ and β_2_ are the first and second independent variables coefficient and the error is assigned by ∈.

(1) $$y=\beta_{o}+\beta_{1}X_{1}+\beta_{2}X_{2}+\beta_{n}X_{n}+\epsilon$$

The polynomial models exhibit a curved shape of response, the second-order polynomials function is used as provided in Equation (2), where y is the measured response, X_i_ and X_j_ are the coded values of independent variables, i and j denote the linear and quadratic equations coefficients, respectively, β_o_ is the y-intercept at X_1_ = X_2_ = 0, k is the number of independent variables and ∈ is the error [66,67].

(2) $$y=\beta_{{}^{\circ}}+\overset{k}{\underset{i=1}{\sum }}\beta_{i}X_{i}+\overset{k}{\underset{i=1}{\sum }}\beta_{ii}X_{j}^{2}+\overset{k}{\underset{j=2}{\sum }}\overset{j=1}{\underset{i=1}{\sum }}\beta_{ij}X_{i}X_{j}+\in$$

The analysis of variance (ANOVA) was carried out in order to determine the significance of the variables using *p*-values [68]. The central and relating variables with *p*-values less than 0.05 were regarded to be substantial in impacting the responses otherwise, it was considered to yield a statistically insignificant effect [68,69]. The obtained optimal variables are validated by compressive strength experiments. To carry out the optimization, Design Expert© software version 11.1.2 was used and the variable criteria were defined in such a way that the lower and higher limits were taken into account to guarantee that all potential combinations were captured, while the response was set to the maximum value, exempting the variable age that was set “in range” because concrete strength increases with age definitely. The data inserted in a proposed model by “user-defined method” were collected from the laboratory tests to achieve optimization using RSM.

The variables were calcination temperature, calcination duration, percentage of replacement, and curing age. The optimization criteria were setting “Minimize” for the calcination temperature and duration, “Maximize” for the percentage of replacement and strength and “In range” for the curing age. The investigated response and variables and their optimization goals were set as presented in Table 1.

## 3. Results and Discussion

### 3.1. Chemical Composition

The chemical components that were analysed using an XRF-spectrometer for the raw FOSA sample and the treated samples are shown in Table 2.

The SiO_2_ content of OPC and untreated ash is 17.6% and 25.5%, respectively, and ranged from 26.0% to 33.4% for the treated samples depending on treatment duration and temperature. This increases the pozzolanic activity due to its high SiO_2_ content [68]. The CaO compounds in treated ash varied between 39.9% and 48.2%. The FOSA samples can be classified as a high lime or class C fly ash as prescribed by ASTM C618–19 [69]. The content of the major components SiO_2_ + Al_2_O_3_ + Fe_2_O_3_ was less than 70%, and the CaO content was more than 10%. LOI must be estimated when using any by-product materials. As it is a magnitude and measure of the residual carbon content, a high value of LOI is known to increase the mix demand for water and superplasticizer [70]. According to ASTM C618 specifications, LOI should not exceed 10% for mineral admixture class N and 6% for classes F and C fly ashes. It can be noticed that increasing the temperature and time of burning was accompanied by a decrease in LOI.

**Figure 2:**
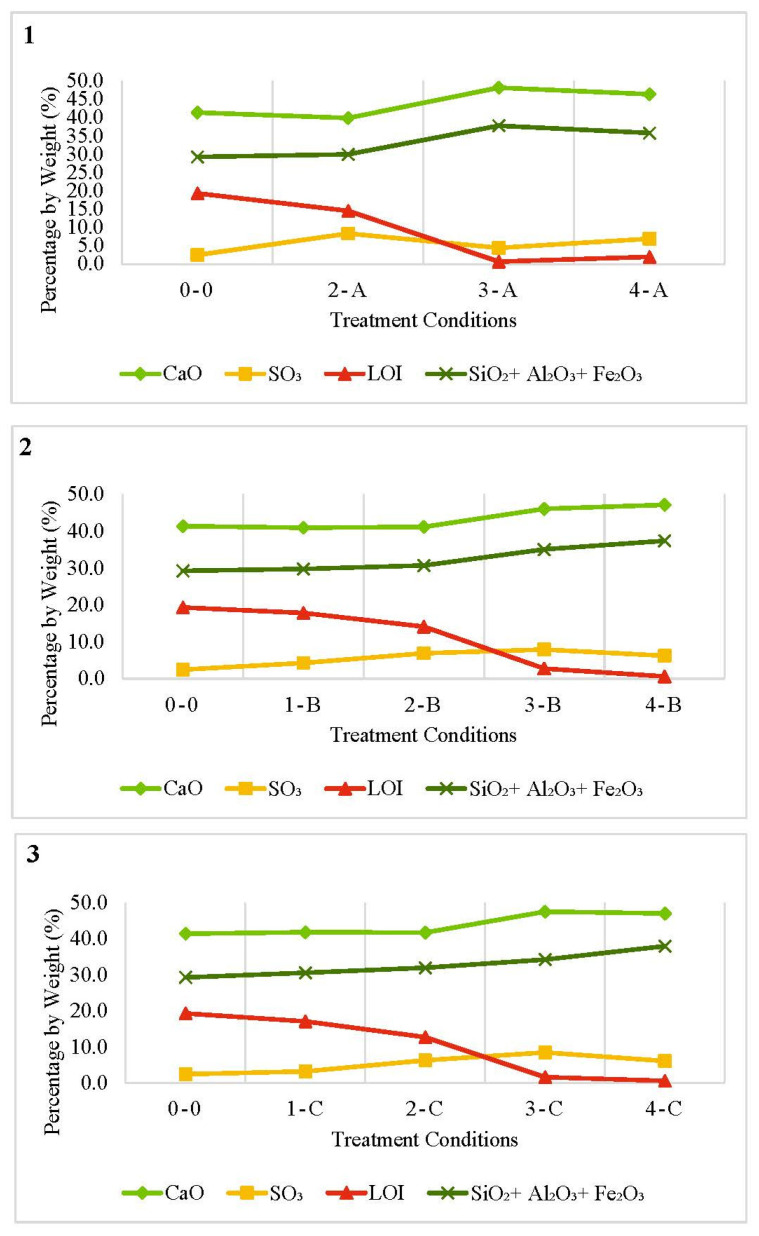
Comparison between CaO, SO_3_, LOI, and (SiO_2_ + Al_2_O3 + Fe_2_O_3_) contents at different temperatures for specific calcination durations: (**1**) Two hours of calcination at 700 °C, 850 °C and 1000 °C, (**2**) Four hours and (**3**) six hours of calcination at 550 °C, 700 °C, 850 °C and 1000 °C.

The effect of the treatment temperature on the FOSA chemical composition and LOI is illustrated in Figure 2 at different heating durations. It was observed that lime (CaO) was the predominant content of FOSA, and this content increased as the treatment temperature increased. A maximum increment of 17.3% was obtained for burning at 850 °C and similar results were obtained for burning at 1000 °C.

The main oxides that were changed during the treatment processes are silica, alumina, and iron oxide. The change was a function of the treatment temperature and duration; however, the changes occurred only at small percentages as the calcination temperature increased. The contents of silica, alumina and iron oxide were in the ranges of 26–33.4%, 2.6–3.3%, and 1.1–1.3%, respectively. The potential hydraulic ability of a pozzolan mainly depends on its reactivity, soluble silica, alumina, and iron oxide content [71]. The amount of silica has indeed been positively enhanced by the treatment process and it was higher by about 3.1% at 1000 °C. In general, a higher treatment temperature results in decreasing the content of all other oxides (e.g., Na_2_O). The removal of these impurities has increased the silica content yield after the burning process.

Following heat treatment from 550 °C to 1000 °C, the color of FOSA changed from dark grey to milky white, as shown in Figure 1. A probable explanation is that the rise in temperature causes an equal spread of heat within the ash resulting in a uniform and efficient transformation of the unburned carbon content into ash particles [72]. The result is the formation of white ash without any leftover carbon that dyes the FOSA with a greyish color. This can be confirmed by observing the reduction of LOI percentages through the heating treatment. On the other hand, the potassium oxide (K_2_O) content that imparts the dark color of FOSA is reduced that also contributed to the white color of FOSA produced after the completion of the burning process [73,74].

The heat treatment significantly increased the content of sulfur oxide (SO_3_). Its content increased three times more than untreated ash, especially at 1000 °C. This is consistent with the results obtained by comparing low- and high-temperature experiments on three different types of fly ash on the formation of SO_3_ [75]. The formation at low temperature is more complicated since it depends on the carbon content and the alkaline materials tend to offset any catalytic effect associated with fly ash. This means that cracking due to dry shrinkage of the material may significantly be improved by a higher SO_3_ content that enhances the cracking resistance, strength development, and volume stability [76,77,78]. However, the SO_3_ content did not comply with the ASTM standard specifications (ASTM C618) when the temperature increased beyond 850 °C.

**Figure 3:**
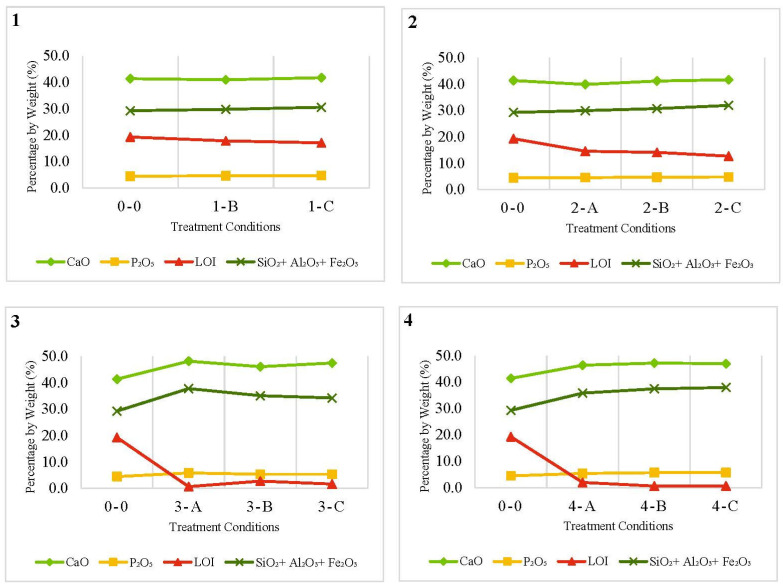
Comparison between (CaO, P_2_O_5_, LOI, and (SiO_2_ + Al_2_O_3_ + Fe_2_O_3_)) contents at different durations for specific calcination temperatures: (**1**) calcination at 550 °C for 4 h and 6 h, (**2**) calcination at 700 °C, (**3**) calcination at 850 °C and (**4**) calcination at 1000 °C for 2 h, 4 h and 6 h.

Regarding the chemical composition characterization from the calcination duration viewpoint, Figure 3 shows a comparison between the tested times at constant temperatures. The total content of SiO_2_ + Al_2_O_3_ + Fe_2_O_3_ and CaO was not significantly affected when changing the treatment duration for the same degree of temperature. Yet, the content of these oxides was slightly increased for a longer duration at all treatment temperatures. As can be seen from the LOI percentages, the treatment durations were insufficient to achieve the complete removal of the carbon content.

Burning FOSA at 850 °C for 2 or 6 h resulted in ash samples with relatively low carbon content. It was also observed that burning at 1000 °C for 6 h or 850 °C for 2 h could both produce a treated FOSA with a low LOI percentage. For both treatment conditions, the LOI percentage was very close and satisfied the ASTM requirements (ASTM618). Thus, to obtain FOSA that complies with LOI percentage as required by ASTM standards for pozzolan and fly ash, the treatment can be conducted at 850 °C and for 2 h only. This will provide acceptable FOSA considering the economy of the treatment process. However, considering other chemical compositions, i.e., SiO_2_ + Al_2_O_3_ + Fe_2_O_3_ content that may affect the cementitious properties of FOSA, burning at 1000 °C for 6 h resulted in a slightly higher content of SiO_2_ + Al_2_O_3_ + Fe_2_O_3_. The increment was 0.5% compared to burning at 850 °C for 2 h.

As discussed previously, the treatment temperature showed significant effects on the color of FOSA, while the duration of treatment did not show any considerable color variation. The LOI and K_2_O did not strongly affect by the treatment duration, which can explain the unchanged color at the same temperature. The increment in phosphorus oxide (P_2_O_5_) was also noticed to be about 28% higher than in the raw FOSA sample while increasing treatment duration up to 6 h at 1000 °C has a harmful effect on the hydration performance of cement and cement-based materials strength. This is blamed on the reduction of SiO_2_ content to increase P_2_O_5_ content which may result in a drop in early strength value as the P_2_O_5_ content increases [79].

### 3.2. X-ray Diffraction Analysis

XRD has been recognized as one of the most adopted techniques for the mineralogical characterization of OS and OSA [80]. XRD analysis is usually performed to identify the degree of crystallinity and amorphousness of the material. The major minerals present in the parent sample are calcite, quartz, anhydrite, fluorapatite, and traces of clay minerals similar to what has been reported in previous studies [4,57,81,82]. Quartz was detected in all samples. It was potentially a result of the high silica (SiO_2_) content in FOSA, and the peak values decreased over time. The XRD patterns show that a remarkable amount of calcite was consumed during the thermal treatment, especially when the calcination temperature was equal to or greater than 850 °C as the reaction in Equation (3) shows [83].

(3) $${\mathrm{CaCO}}_{3\left(S\right)}\;\overset{\Delta }{\to }{\;\mathrm{CaO}}_{\left(S\right)}+{\mathrm{CO}}_{2}$$

The reactions between portlandite and silica are known to produce calcium silicate hydrate (C-S-H) that is responsible for the strength development in cementitious materials. These reactions represent the pozzolanic activity of the material [84,85]. According to the pozzolanic activity index, there are four mineral admixture classes [86]. The first type is the high-active pozzolans and mainly consists of pure silica in a non-crystalline form. The second type is normal pozzolans, which mostly consist of silicate glass with aluminum, iron, and alkalis, in addition to a small quantity of crystalline matter commonly containing quartz, mullite, sillimanite, hematite, and magnetite. The third type is natural materials such as natural pozzolans that mainly contain quartz, feldspar, and mica. The last type is weak pozzolan materials, which essentially comprise crystalline silicate minerals and a slight amount of non-crystalline matter. Generally, the amorphous state of a material is considered an indication of reactive silica (SiO_2_) [87].

**Figure 4:**
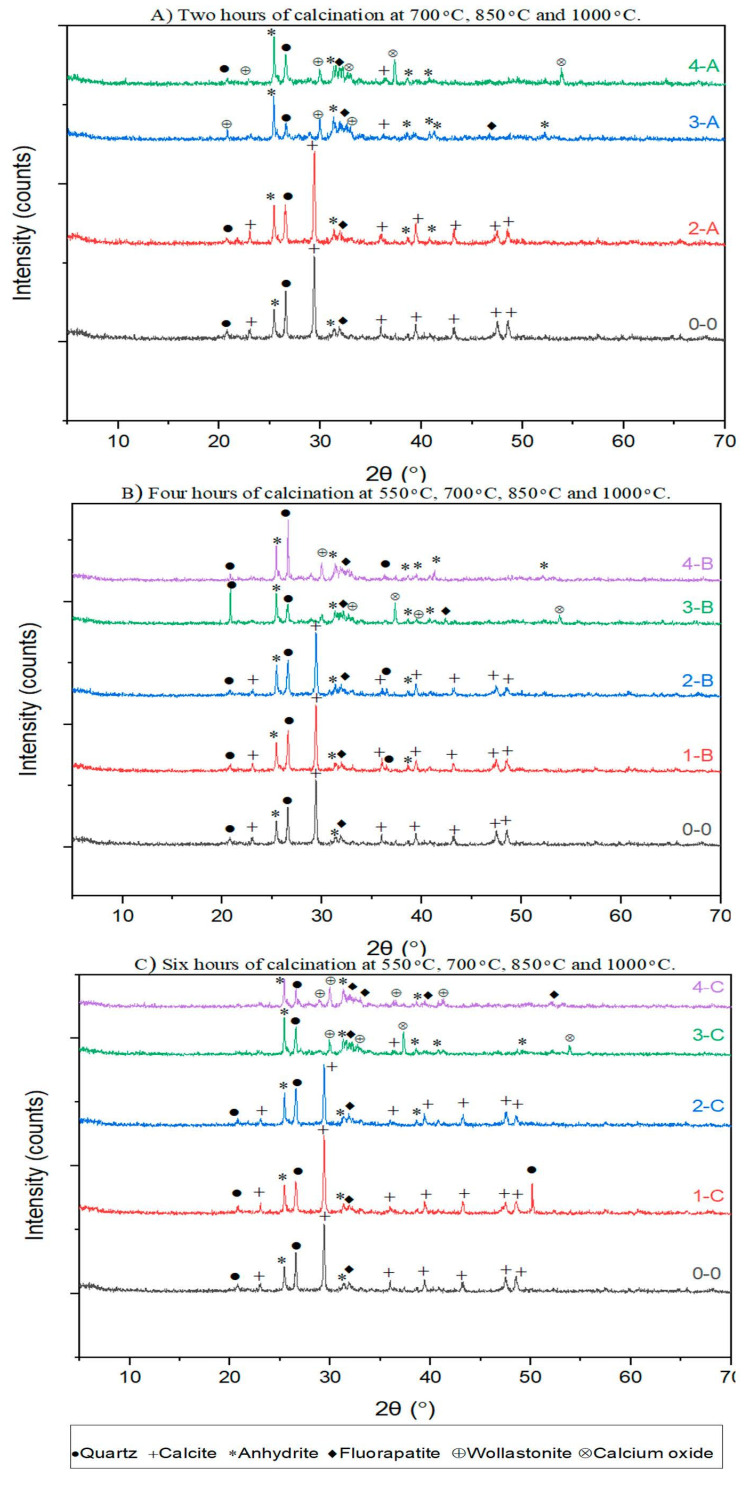
Comparison between XRD- patterns for ash treated at different temperatures with specific calcination durations.

Considering the pozzolanic reactivity of materials has a linear relation with their amorphous phases, understanding the pre-treatment effect on crystalline phases of FOSA comprehensively is essential. There are several factors, such as surface area, particle size, carbon content, and LOI, which affect the pozzolanic activity of FOSA, and thereby have a meaningful impact on the mechanical engineering properties. Figure 4 depicts the impact of thermal treatment on XRD patterns for raw and treated samples.

For all treatment durations, the XRD patterns confirm that the thermal treatment up to 750 °C does not increase or change the crystalline structures of FOSA. It can be noted that calcite, quartz, and anhydrite were the major crystalline phases due to the low-temperature treatment, while fluorapatite was the minor crystalline phase. Accordingly, by increasing the calcination temperature, the phases of fluorapatite and calcite are indirectly switched, where calcite became the minor phase since it is thermally decomposed. As a result of the thermal treatment, crystal phases such as calcium oxide, wollastonite, and anhydrite develop. On the other hand, when increasing the temperature up to 1000 °C, calcite was decomposed, and the decomposition is accompanied by chemical reaction and phase transformation during cooling. This transformation in phases can be recognized simply by comparing XRD patterns of 550 °C and 1000 °C.

**Figure 5:**
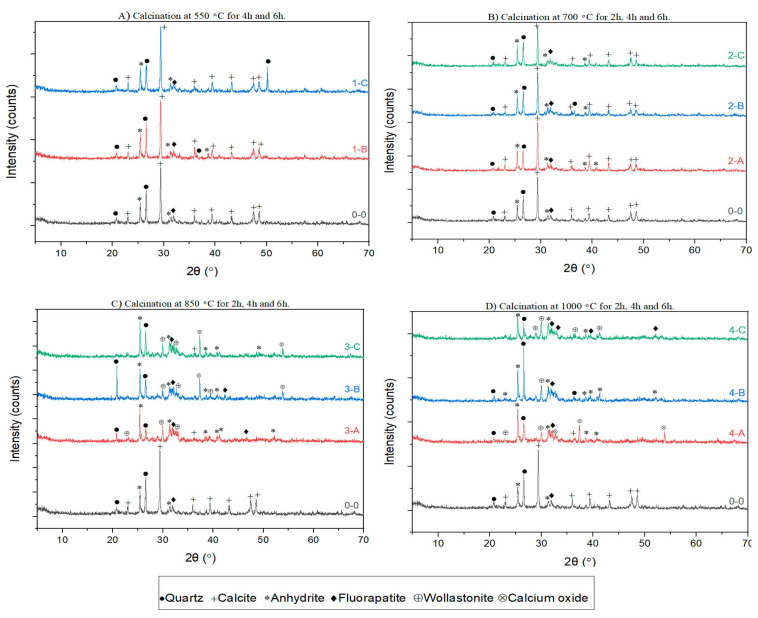
Comparison between XRD- patterns for ash treated at different durations for specific calcination temperatures.

The effect of calcination durations on the XRD patterns of treated FOSA at temperatures of 550 °C, 700 °C, 850 °C, 1000 °C is clarified in Figure 5. The patterns clearly show that increasing the duration of pre-treatment at 550 °C and 700 °C (low heat levels) slightly affected the crystallization process. This is credited to the crystalline transformation process to other structures that require the absorption of energy [83]. The calcination for 2 h was enough to convert completely the quartz of FOSA into soluble materials. Also, it can be observed that calcite is the most stable thermodynamically under external conditions, which agrees with the literature [88]. The patterns reveal that the calcite peaks at 2θ angles were about 25.4° to 29.3° at it directly decomposed by increasing the temperature and duration of the treatment. At temperatures of 850 °C and 1000 °C for 6 h, the XRD pattern of the FOSA samples indicated the existence of an amorphous state.

The FOSA samples reveal a cementitious behavior due to the pozzolanic content presented by (SiO_2_ + Al_2_O_3_ + Fe_2_O_3_), and alkali content provided by CaO. The strength development in all samples is linked to reactions of CaO with the pozzolanic components for C-S-H and calcium aluminate hydrate (C-A-H) formulation. Sulphate minerals (ettringite, anhydrite, and gypsum) are likely to form due to SO_3_ availability in the FOSA as recognized by the XRD diffractograms.

**Figure 6:**
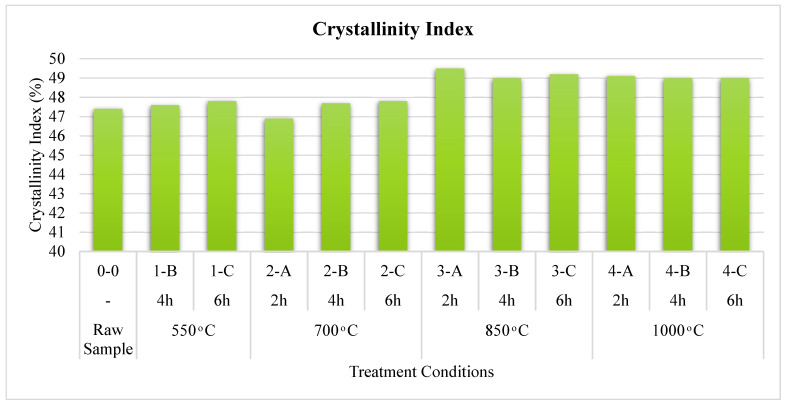
Relation between crystallinity index and treatment conditions for FOSA samples.

To provide extra analysis concerning the relative differences between samples, the XRD peak height technique was used to compute the crystallinity index as per Equation (4) [89], and the result is summarized in Figure 6.

(4) $$\mathrm{Crystallinity}\;\mathrm{index}=\left(\frac{\mathrm{Area}\;\mathrm{of}\;\mathrm{Crystalline}\;\mathrm{Fraction}}{\mathrm{Area}\;\mathrm{of}\;\mathrm{Crystalline}\;\mathrm{Fraction}\;+\;\mathrm{Area}\;\mathrm{of}\;\mathrm{Amorphous}\;\mathrm{Fraction}\;}*100\%\right)$$

The results indicated a minor change in the crystallinity indexes at low temperatures. The potential effect at high temperatures above 850 °C can be attributed to the chemistry of ash-like CaO content. The effect of burning duration has a gradual increase in crystalline content starting from 2 h up to 6 h for low temperatures, the highest strength growth was 4.4% above the strength of the raw sample for 2 h calcination at 850 °C, and then became almost the same at 1000 °C.

### 3.3. FOSA-Based Mortar Compressive Strength

**Figure 7:**
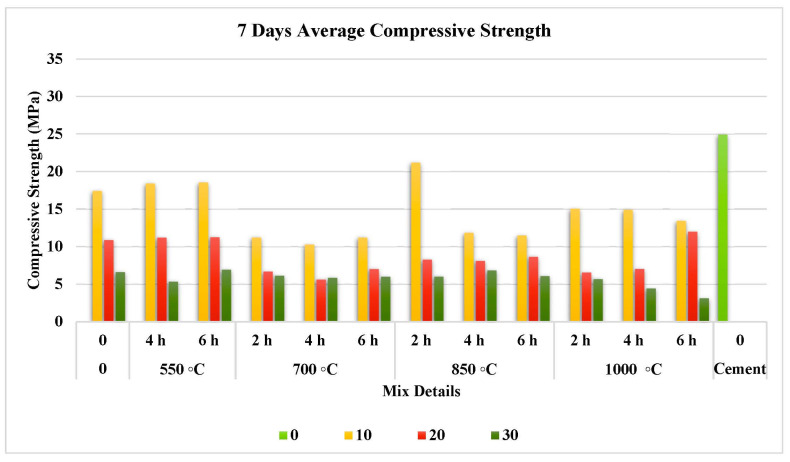
Effect of pre-treatment on 7 day compressive strength.

**Figure 8:**
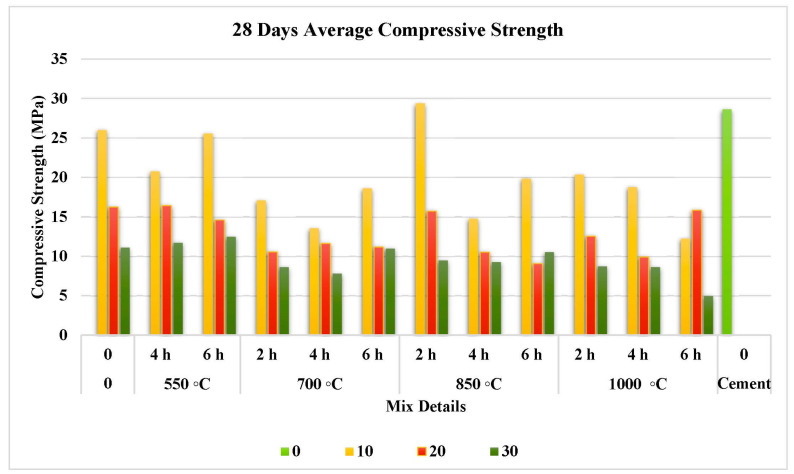
Effect of pre-treatment on 28 day compressive strength.

**Figure 9:**
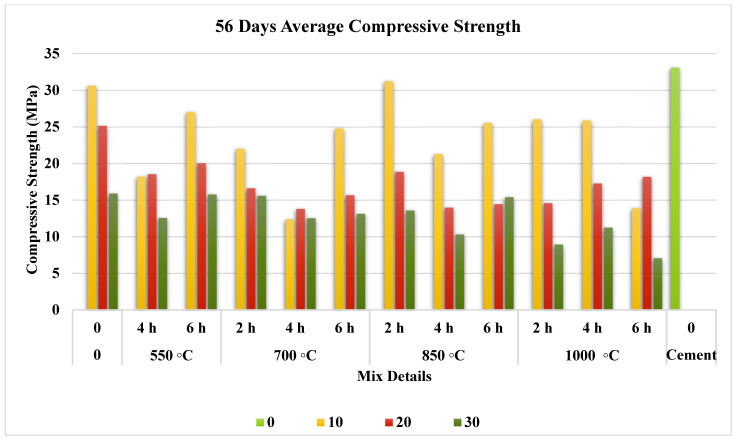
Effect of pre-treatment on 56 day compressive strength.

The compressive strength test results at 7, 28, and 56 days are illustrated separately in Figure 7, Figure 8 and Figure 9. They prove that the addition of FOSA led to a reduction in early strength and this reduction has increased as the replacement ratio increased.

#### 3.3.1. Effect on Short-Term Compressive Strength

Figure 7 shows the influence of utilizing FOSA in both cases before and after treatments on the 7 day compressive strength of mortar specimens. Compressive strength increases during the early curing stage as calcination temperature increases. However, regardless of the maximum compressive strength at the maximum temperature or duration of treatment, the heat treatment impact declines as calcination time and temperature increase. In comparison with the reference specimens, the untreated FOSA recorded a 30% reduction in strength. The reduction for treated FOSA ranges from 11.9% to 87.5%. This wide range may be attributed to the effect of treatment on the activity of FOSA, crystalline structure, and replacement ratio.

The highest compressive strength at this age occurred for 10% of cement substitution, with only an 11.9% reduction in strength for FOSA that was calcined at 850 °C for 2 h, which matches the mineralogical and morphological analysis of this case of treatment. Moreover, the 20% and 30% replacement ratios lead to a tremendous decrease in strength, which reached more than 80% in the mix treated at 1000 °C for 6 h with 30% replacement of cement. This might be attributed to the progressive nature of the pozzolanic reactions between the glassy crystalline particles in the ash with the CH produced during the hydration process. Compared to the raw samples, the thermal treatment demonstrated increased strength by up to 21.78% and 5% and down to for 3-A (10%) and 2-A (20%). The treatment for 2 h is the most effective economically and practically.

#### 3.3.2. Effect on Medium-Term Compressive Strength

Considering the results in Figure 8, the strength of all mixes increased at 28 days when compared with the 7 day results and can increase further at later ages. This can be linked to the longer time of curing that improves the chance for the alkalis in ash to form an extra cementitious matrix when reacting with the silica. Except for 1000 °C, the strength was noticed to rise at minimal calcination duration at all applied treatment temperatures and to decrease when the calcination time of treatment increased.

The treated FOSA sample at 850 °C for 2 h with replacement at 10% gave around 13% and 3% higher strength compared with the reference and cement mortar specimens, respectively. All the remaining samples showed a decrement in strength compared to the untreated samples, 1-B (20%), 1-B (30%), and 1-C (30%), which showed a slight increase in strength but still lower that of cement (target strength). The reduction in compressive strength is attributed to CaCO_3_ amount that reacts with cement hydrates, which limits the pozzolanic activity for intermediate and long-term effects [90]. Replacement of cement with FOSA by up to 10% gave a higher compressive strength while exceeding this level of replacement reduced the compressive strength as compared to the cement mortar. In addition, increasing the calcination duration decreased the strength dramatically whatever the temperature and ratio of replacement.

#### 3.3.3. Effect on Long-Term Compressive Strength

All cured FOSA samples exhibited similar performance to that of the hydrated cement products, but with a lower level of compressive strength as seen in Figure 9. The effect of the replacement ratio does not change, as well as the calcination duration. The strength improvement from 28 to 56 days is estimated to have a minor effect because of the continuous decrease in CaO content in the mix as the hydration reactions continue, which is directly proportional to the power of the ash–alkalis–silica reaction [91]. The compressive strength result of the non-treated ash samples gave a strength of 30.6 MPa versus 33.09 MPa for cement samples, while the sample containing ash calcined at 850 °C for 2 h showed 31.21 MPa strength. This improvement is due to the superior hydration and pozzolanic reactivity, where more SiO_2_ and Al_2_O_3_ react with CaSO_4_ and CaO. This hydration reaction results in an improvement in compressive strength [45]. Overall, the compressive strength results are considered satisfactory for the use of FOSA in several types of construction materials.

#### 3.3.4. Pozzolanic Strength Activity Index

**Figure 10:**
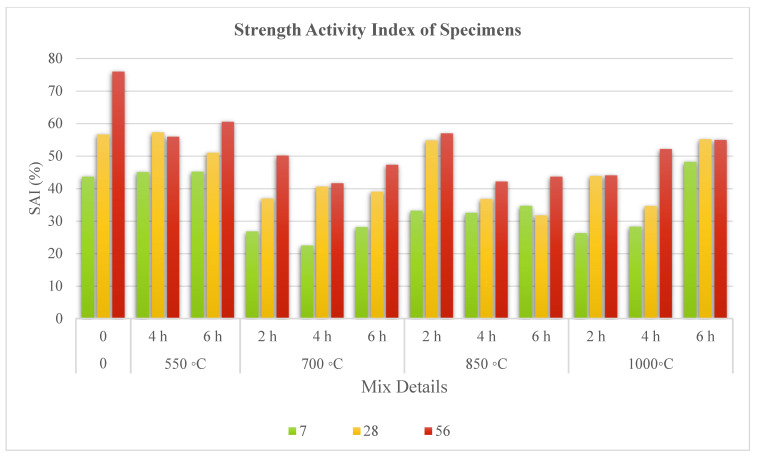
Effect of pre-treatment on strength activity index of samples containing 20% ash as cement re-placement.

The strength activity index (SAI) for all mortar samples was calculated based on ASTM C311 specification at 20% cement replacement and the results appear in Figure 10. SAI represent the FOSA mortar strength to reference (cement mortar) strength ratio at each curing period (age of sample). The pace of cement mortar strength growth is mostly determined by its hydration rate. [92]. In contrast, this rate depends on the hydration and rehydration of cement produced by the pozzolanic reactivity of FOSA in the mortar.

The SAI at 56 days was above the minimum specifications as defined in ASTM C 618. The non-treated FOSA provided a SAI of 43.6%, 56.6%, and 75.9% of that of the reference specimens’ strength at 7, 28, and 56 days, respectively. At the early ages of 7 and 28 days, substituting cement with 20% of treated FOSA proved to decrease the compressive strength in contrast to the reference cement mortar. This is associated with the dilution effect and late-onset pozzolanic response of ash with Ca(OH)_2_. The compressive strength of mortar with ash calcined at 550 °C was higher than that of other mortars at most ages. This could be attributed to the change in quantity and kind of amorphous phase. These results generally agree with the experimental outcomes of Bourdot [45], who found that, before 7 days of curing time, the mixes with 20% of POFA had lower compressive strength than those with 10% of POFA. However, the combustion duration of 2 h is also ideal to produce high activity FOSA.

### 3.4. Statistical Analysis

#### 3.4.1. Analysis of Variance

The ANOVA findings for the measured responses (compressive strength (σ)) of the FOSA mortar samples are summarized in Table 3. The created model’s F-value of 32.40 indicates that it is significant, with just a 0.01% chance that an F-value this big could arise due to noise. Values of “Prob > F” less than 0.05 or greater than 0.1 mean the significance or insignificance of the model terms, respectively.

The analysis revealed that B, C, D, AC, A^2^, B^2^, and D^2^ were the significant model terms that contributed to the improvement of the model. A significant interaction (one variable could affect the result of the other variable) between investigated parameters was not observed, where the *p*-value for most of the interaction terms was less than 0.05 except for the term AC. Thus, the temperature degree affected the replacement ratios. All of the Variance Inflation Factor (VIF) values are equal to 1, thus indicating multicollinearity does not exist and that the variables were orthogonal. The compressive strength model was developed after removing the irrelevant terms, excluding those required to maintain model hierarchies and their interactions. The final compressive strength model of FOSA-based mortar comprising all terms are shown in Equation (5).

(5) $$\mathrm{Ln}\left(\sigma \right)=2.57-0.056B-0.37C+0.33D-0.12AC-0.14A^{2}+0.099\;B^{2}-0.11\;D^{2}$$

Table 4 shows the properties of the developed model for compressive strength of FOSA-based mortar. The quality, fitness, and adequacy of the developed compressive strength model were validated through the degree of correlation (R^2^). R^2^ value indicates that the model has a high correlation degree where 85% of the experimental values of compressive strength can be represented by the developed model. The low variance value (0.0395) between the adjusted R^2^ (Adj. R^2^) and predicted R^2^ (Pred. R^2^) for the model signs reasonable conformity between them. Furthermore, the standard deviation (SD) of the model was employed to judge the variability of the experimental set of data. The low value of the SD compared with the mean value tells that the fitting tendencies between the experimental and model predicted data have a high correlation degree. The adequacy precision (AP) value of the model was higher than 4, indicating that the developed model can be used to navigate the design space.

**Figure 11:**
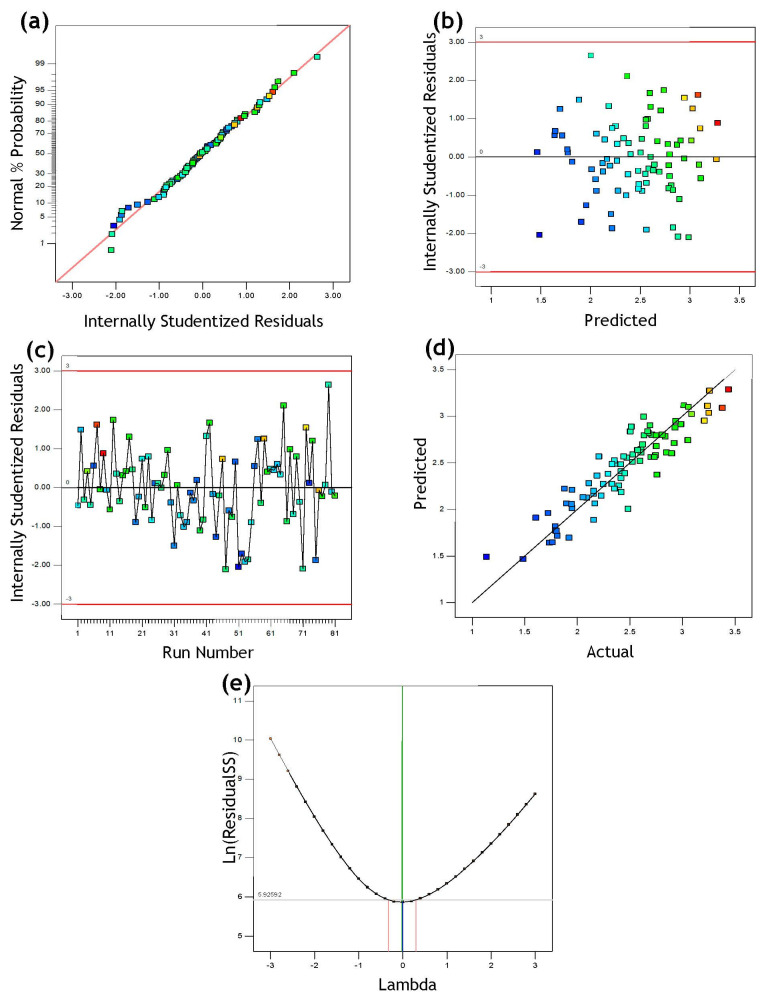
The response model diagnostic plots: (**a**) normal residuals, (**b**) residual vs. predicted, (**c**) residuals vs. run, (**e**) predicted vs. actual, and (**d**) Box-cox.

The model was also examined for its adequacy by the diagnostic plots, as displayed in Figure 11. These plots are also highly important for model verification. From the normal residual plot, the normal distribution of the data can be extracted whereas the data points are ranged parallel to the inclined straight line. The residuals vs. expected diagram show that the residuals values were distributed in a random and consistent way below and above the reference line, due to uniform data variation. The box-cox plot for power transform shows that the power transformed is needed since the value of lambda was less than 1. However, further studies showed that the transformation effect can be neglected when the ratio of the maximum to minimum values is less than 3 [93]; so, no transformation was required for the developed model.

Finally, the plot of predicted versus actual compressive strength shows that the values are almost aligned lengthways along the inclined straight line, which indicates an adequate accuracy of the values predicted by the optimized model. The response of the developed model is sufficient and adequate to estimate the FOSA-based mortar compressive strength.

#### 3.4.2. Contours Plots of Response Surface

**Figure 12:**
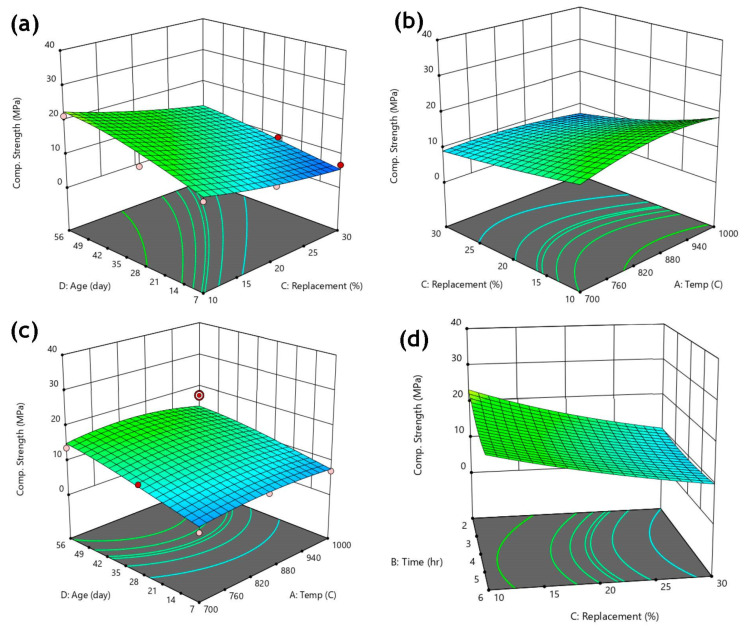
Response surface 3-dimensional contour plots to: (**a**) percentage of replacement vs. curing age, (**b**) calcination temperature vs. percentage of replacement, (**c**) calcination temperature vs. curing age, (**d**) percentage of replacement vs. calcination time.

The 3-dimensional response surface plots of FOSA-based mortar compressive strength and the investigated parameters are shown in Figure 12. The response (compressive strength) was expressed as two independent variables based function at a period with compressive strength predicted.

The plots show the existence of an optimum value for each parameter except for the replacement ratios with age as indicated by the almost flat-sloped surface of the response in Figure 12a. Figure 12b demonstrates an increase in compressive strength at a temperature of around 880 °C with an optimal replacement at about 10% after further temperature increases reduced the predicted compressive strength. FOSA-based specimens reached the optimal compressive strength at 10% cement replacement, a temperature of 880 °C, and 2 h of calcination and increased with age. This implies that the FOSA pozzolanic activity was enhanced by the curing (age) effect. The curves become steeper as increasing the temperature results in constantly decreased strength. This proves the sensitivity of FOSA-based samples to compressive strength variations.

#### 3.4.3. Response Optimization

The most critical element to achieving sustainability is optimization, the purpose of optimization is to find the best viable solution while keeping the issue limitations into consideration [94]. The optimization criteria settings vary by adopting different amounts and conditions, based on the required settings in order to achieve the wanted purpose. The desirability function was used in this study to generate that more than one optimization criterion is considered at the same time. One of the most extensively utilized strategies in the industry for optimizing multiple response processes was developed by Derringer and Such [95]. As seen in Equation (6), the approach employs an objective function known as the desirability function (D), which converts an estimated response into a scale-free value known as desirability (di) with weight importance factor (w); the factor settings with the highest overall attractiveness are regarded to be the best parameter conditions [96].

(6) $$D=\left(d_{1}^{\omega_{1}}\times d_{2}^{\omega_{2}}\times \ldots \times d_{n}^{\omega_{n}}\right)\;\;$$

The optimized solution from the used software of the independent variables in the study was obtained at 30% cement replacement, after 700 °C burning for 2 h to achieve a compressive strength of 13.175 MPa after 56 days of curing with a desirability value of 88.9%. These conditions made the utilization of OSA more sustainable and keep costs down since less treatment is required to upgrade the final product.

#### 3.4.4. Model Validation

To verify the model accuracy, the optimum result predicted values were used to perform a laboratory experiment to examine the experimental and predicted results by measuring the absolute relative deviation (ARD, %) as presented in Equation (7) [97]. The average ARD value for the three tested specimens was 7.4%. It can be concluded that the model predicted the response with acceptable accuracy.

(7) $$\mathrm{ARD}\left(\%\right)=\frac{Experimental\;results-Predicted\;reults}{Experimental\;results}\times 100$$

## 4. Conclusions

The following conclusions can be drawn from this study:

- The results showed that the heat treatment has influenced the pozzolanic potential of FOSA. The effect of the calcination temperature was more preponderant on the XRF and XRD results. The chemical composition of FOSA varied according to the temperature and duration of combustion. All of the ash samples were essentially composed of CaO. The variability in SiO_2_ and CaO content was a result of LOI variation. All tested samples have an approximately similar chemical composition, and are similar to the raw constituents used in cement production;
- Grinding and calcination were effective in removing organic matter, but they cannot be used as the exclusive method to boost FOSA pozzolanic activity, because even after these treatments the material does not reach an appropriate significant reactivity. The pozzolanic activity of FOSA was observed to be closely correlated to the calcination temperature. The pozzolanic activity of FOSA could not be adequately stimulated via calcination at 550 °C. As a result, the effective heat activation temperature for FOSA was around 850 °C. In contrast, in the SAI with cement test, the difference in the pozzolanic activity between treated and non-treated ashes was greater. This is due to the higher filling effect. The raw ash reached the standard values required for classifying the material as a pozzolan material based on the compressive strength requirements. This opens the opportunity to investigate other types of upgrading processes;
- The results of compressive strength are considered suitable to be used in several construction materials in which low strength is required. The increment in the percentage of replacement reduces the strength of the produced mortar at all levels of treatment. A curing age of 28 days and more improves the compressive strength and solved delays in the activity and the dilution effect. The maximum compressive strength at all ages was recorded at 850 °C calcination temperature for 2 h with 10% replacement;
- The developed model using RSM was effectively used to evaluate the compressive strength of FOSA-based mortar. The ANOVA results confirmed the significance of all investigated parameters. The optimum responses were obtained at 30% cement replacement, 700 °C calcination temperature for 2 h, and 56 days of curing. The developed optimization technique assists in determining the balance for getting eligible properties.

The collaboration of this type of waste material with cement is a very promising sustainable improvement. However, high levels of replacement affect the quality and strength of concrete. Future studies concern the process of chemical treatment, increasing the interfacial transition zone binding by densifying the structure, and cost analysis. In addition, a comparison between types of OS from different parts of the world is needed to identify the characteristics that distinguish each type in order to reach greater enhancement.

## Figures and Tables

**Table 1:** Criteria settings for optimization.

Designation	Factor/Response	Unit	Goal
A	Temperature	°C	Minimize
B	Time	Hours	Minimize
C	Replacement	%	Maximize
D	Age	Days	In range
σ	Compressive strength	MPa	Maximize

**Table 2:** Chemical compositions of raw, OPC and treated FOSA (% by weight).

	Calcination Temperature											Raw Sample	OPC	
	550 °C		700 °C			850 °C			1000 °C					
Calcination duration	4 h	6 h	2 h	4 h	6 h	2 h	4 h	6 h	2 h	4 h	6 h	-	-	
Designation	1-B	1-C	2-A	2-B	2-C	3-A	3-B	3-C	4-A	4-B	4-C	0-0	OPC	
Oxide (wt.%)														
SiO_2_	26.00	26.80	26.30	26.90	28.00	33.20	30.80	30.00	31.40	32.90	33.40	25.50	17.60	
Al_2_O_3_	2.65	2.63	2.56	2.68	2.76	3.32	3.07	3.02	3.15	3.24	3.28	2.66	4.02	
Fe_2_O_3_	1.14	1.15	1.11	1.15	1.19	1.29	1.23	1.22	1.25	1.29	1.28	1.13	3.19	
CaO	41.00	41.80	39.90	41.20	41.70	48.20	46.10	47.50	46.40	47.20	47.00	41.40	66.41	
MgO	1.01	1.03	0.97	1.01	1.03	1.24	1.16	1.20	1.28	1.22	1.22	1.08	1.35	
Na_2_O	0.00	0.06	0.00	0.00	0.00	0.00	0.00	0.00	0.41	0.00	0.00	0.43	0.02	
K_2_O	0.36	0.36	0.35	0.37	0.37	0.34	0.37	0.34	0.45	0.33	0.30	0.44	0.40	
SO_3_	4.28	3.25	8.37	6.92	6.31	4.44	7.94	8.49	6.91	6.25	6.11	2.49	4.17	
P_2_O_5_	4.62	4.69	4.56	4.71	4.81	5.88	5.36	5.36	5.42	5.70	5.73	4.49	0.08	
F	0.30	0.31	0.62	0.38	0.45	0.49	0.32	0.48	0.46	0.41	0.41	0.39	0.00	
MnO	0.01	0.01	0.01	0.01	0.01	0.01	0.01	0.01	0.01	0.01	0.01	0.01	0.19	
LOI	17.84	17.11	14.55	14.12	12.72	0.67	2.77	1.67	1.97	0.63	0.62	19.33	3.10	
SiO_2_ + Al_2_O_3_ + Fe_2_O_3_	29.79	30.58	29.97	30.73	31.95	37.81	35.10	34.24	35.80	37.43	37.96	29.29	24.81	

**Table 3:** ANOVA for response surface quadratic of compressive strength model.

Source	Sum of Squares	df	Mean Square	F-Value	*p*-Value
Model	15.00	12	1.25	32.40	<0.0001
A	3.844 × 10^−^³	1	3.844 × 10^−^³	0.100	0.7532
B	0.17	1	0.17	4.37	0.0402
C	7.28	1	7.28	188.68	<0.0001
D	6.00	1	6.00	155.41	<0.0001
AB	0.099	1	0.099	2.56	0.1143
AC	0.49	1	0.49	12.83	0.0006
AD	0.045	1	0.045	1.17	0.2838
BC	0.036	1	0.036	0.94	0.3354
CD	0.14	1	0.14	3.65	0.0605
A^2^	0.35	1	0.35	8.98	0.0038
B^2^	0.18	1	0.18	4.57	0.0362
D^2^	0.22	1	0.22	5.81	0.0186
Residual	2.62	68	0.039		
Cor Total	17.62	80			

**Table 4:** Evaluation of the selected model for compressive strength.

R^2^	Pred. R^2^	Adj. R^2^	Adj. R^2^-Pred. R^2^	SD	AP	Mean
0.8512	0.7854	0.8249	0.0395	0.20	23.101	2.45

## Data Availability

The data used to support the findings of this study are available from the corresponding author upon request.
